# A nutritionally-enhanced oil from transgenic Camelina sativa effectively replaces fish oil as a source of eicosapentaenoic acid for fish

**DOI:** 10.1038/srep08104

**Published:** 2015-01-29

**Authors:** M. B. Betancor, M. Sprague, S. Usher, O. Sayanova, P. J. Campbell, J. A. Napier, D. R. Tocher

**Affiliations:** 1 Institute of Aquaculture, School of Natural Sciences, University of Stirling, Stirling FK9 4LA, United Kingdom; 2 Department of Biological Chemistry and Crop Protection, Rothamsted Research, Harpenden AL5 2JQ, United Kingdom; 3 Biomar Ltd., North Shore Road, Grangemouth FK3 8UL, United Kingdom

## Abstract

For humans a daily intake of up to 500 mg omega-3 (n-3) long-chain polyunsaturated fatty acids (LC-PUFA) is recommended, amounting to an annual requirement of 1.25 million metric tonnes (mt) for a population of 7 billion people. The annual global supply of n-3 LC-PUFA cannot meet this level of requirement and so there is a large gap between supply and demand. The dietary source of n-3 LC-PUFA, fish and seafood, is increasingly provided by aquaculture but using fish oil in feeds to supply n-3 LC-PUFA is unsustainable. Therefore, new sources of n-3 LC-PUFA are required to supply the demand from aquaculture and direct human consumption. One approach is metabolically engineering oilseed crops to synthesize n-3 LC-PUFA in seeds. Transgenic *Camelina sativa* expressing algal genes was used to produce an oil containing n-3 LC-PUFA to replace fish oil in salmon feeds. The oil had no detrimental effects on fish performance, metabolic responses or the nutritional quality of the fillets of the farmed fish.

Omega-3 (n-3) long-chain polyunsaturated fatty acids (LC-PUFA) are essential or conditionally essential dietary nutrients for vertebrates with well-established health benefits in humans^1^. Specifically, n-3 LC-PUFA including eicosapentaenoic acid (EPA; 20:5n-3) and docosahexaenoic acid (DHA; 22:6n-3) have key roles in neural development, immune and inflammatory responses, and beneficial effects in several pathological conditions, including cardiovascular and neurological diseases, and some cancers^2^,^3^,^4^,^5^,^6^,^7^,^8^. The International Society for the Study of Fatty Acids and Lipids recommends a daily intake of 500 mg of n-3 LC-PUFA (EPA + DHA) for optimum cardiovascular health^9^ and, projecting this to a population of 7 billion, this amounts to a total annual requirement for over 1.25 million metric tonnes (mt) of n-3 LC-PUFA. The annual global supply of fish and fish oil cannot meet this level of requirement for n-3 LC-PUFA and so there is a large gap between supply and demand^10^.

Microalgae in the marine and aquatic environments are the primary producers of the vast majority of n-3 LC-PUFA^11^, which accumulate in the marine food web and thus fish and seafood are the predominant source of these essential nutrients in the diet^12^. However, global fisheries are at, or beyond, exploitable limits and cannot increase to satisfy the growing demand for fish and seafood^13^ and, therefore, around 50% is now farmed^14^. Paradoxically, high levels of n-3 LC-PUFA in farmed fish and shrimp was only assured by the use of fishmeal and, especially, fish oil, themselves finite and limited marine resources derived from wild fisheries^15^,^16^,^17^. The only biological alternatives to fish oil, vegetable oils, do not contain n-3 LC-PUFA as this biosynthetic pathway is not present in terrestrial plants^18^. Therefore, as demand for fish and seafood increases, so will that of n-3 LC-PUFA and, as supplies are finite^10^, the gap between the two will only continue to increase in the future.

The only sustainable solution to the ever-increasing global demand for n-3 LC-PUFA is *de novo* production of entirely new sources^19^. This requires the application of modern biotechnology ranging from genetic modification through to synthetic biology to introduce the n-3 LC-PUFA biosynthesis trait into appropriate oleaginous platforms^20^,^21^. This has been applied to produce EPA in the yeast *Yarrowia lipolytica*^22^, and DHA in the diatom *Phaeodactylum tricornutum* that normally produces only EPA^23^. However, oilseed crops dominate world oil production and there is a highly organised and well-established infrastructure for the cultivation, harvest, processing, distribution, marketing and utilisation of vegetable oils^24^. Therefore, a highly practical approach to developing a novel, renewable supply of n-3 LC-PUFA is the metabolic engineering of oilseed crops with the capacity to synthesize these bioactive fatty acids in seeds^25^,^26^. Production of EPA and DHA in seeds was initially demonstrated in the model plant *Arabidopsis*^27^,^28^, and was recently reported in an oilseed crop, *Camelina sativa*^29^,^30^. *C. sativa* or false flax, is a member of the Brassicaceae family and an ancient oilcrop that in the wild type produces an oil with up to 45% of fatty acids as α-linolenic acid (LNA; 18:3n-3)^31^.

In the present study, *C. sativa was* transformed with a suite of five microalgal genes to produce a higher plant source of n-3 LC-PUFA^30^. The extracted seed oil, containing 20% of total fatty acids as EPA, was investigated as a replacement for marine fish oil in feeds for Atlantic salmon (*Salmo salar*). Triplicate groups of salmon were fed one of three experimental diets containing fish oil (FO), wild-type Camelina oil (WCO) or EPA-Camelina oil (ECO) as the sole added lipid source for 7-weeks. The results showed that growth performance, feed efficiency, fish health and nutritional quality in terms of EPA + DHA for the human consumer were all unaffected by replacing FO with ECO. Metabolic analysis confirmed the EPA to DHA pathway was active in liver and transcriptomic analysis indicated that the EPA:DHA ratio had greater influence on gene expression than absolute level of EPA. This translational research has demonstrated that n-3-LC-PUFA enriched oils from transgenic oilseed crops can be effective substitutes for fish oil in feeds for Atlantic salmon, capable of maintaining n-3 LC-PUFA levels in farmed fish. Thus, oils extracted from modified oilseed crops represent a potential solution to supplying the growing demand for these critically important dietary nutrients.

## Results

### Seeds from transgenic *Camelina sativa* effectively accumulated over 20% of EPA

The analysis of fatty acid composition of the oil (abbreviated to Tr-CO, for transgenic Camelina oil) extracted from the seeds of transgenic Camelina demonstrated accumulation of significant levels of EPA (>20%; Table 1), as high as those found in many fish oils. This increased the total n-3 PUFA content of Tr-CO to a level similar to fish oil and was accompanied by a reduction in 18:3n-3 compared to the wild-type Camelina oil (Wt-CO). As expected, no DHA was detected in the terrestrial-origin oils. Monoenoic fatty acids, particularly 18:1n-9 and 20:1n-9 were reduced in Tr-CO compared to Wt-CO, but total n-6 PUFA were increased due to the increased percentages of 18:3n-6, 20:3n-6 and arachidonic acid (ARA; 20:4n-6).

### Fatty acid compositions of feeds reflected the lipid source utilised in the formulations

In general terms, the vegetable oil-based feeds, WCO and ECO, had decreased proportions of saturated fatty acids (especially 14:0 and 16:0), 16:1n-7, 18:1n-7 and n-3 PUFA (18:4n-3, 22:5n-3 and 22:6n-3) and increased levels of 18:3n-3, 20:1n-9, 18:2n-6, 20:2n-6 and total n-6 PUFA compared to the FO feed (Table 1). Furthermore, the ECO feed had increased percentages of n-3 PUFA (20:4n-3, 20:5n-3), n-6 PUFA (18:2n-6, 18:3n-6, 20:3n-6, and 20:4n-6) and saturated fatty acids, and decreased monounsaturated fatty acids (18:1n-9, 20:1n-9) in the ECO feed compared to the WCO feed (Table 1). In particular, the ECO feed displayed an EPA level of almost 19% of total fatty acids, similar to that of FO (~16%), whereas EPA content in the WCO feed was just over 2%, being derived from fishmeal employed in the formulation of the experimental feeds. Furthermore, around 2.5% DHA was present in both WCO and ECO feeds (as a result of the addition of fishmeal).

### There was no difference in growth performance and feed efficiency between fish fed ECO and FO

At the end of the trial, fish from all dietary treatments more than doubled their weight and there were no mortalities (Table 2). There were no significant differences in final weight, total length or specific growth rate between fish fed the different diets. Other fish performance and basic health indicators such as hepatosomatic and viscerosomatic index (HSI and VSI respectively) and Fulton's condition factor (k) were unaffected. Feed intake and feed efficiency, as determined by feed conversion ratio, were also not affected by dietary treatment (Table 2). Proximate analysis of whole fish showed that fish fed the ECO diet had similar protein, dry matter and ash compositions to fish fed the FO diet, but lipid contents were higher in fish fed the ECO and WCO diets compared to FO-fed fish (Table 3). Fish muscle (flesh) lipid content was not affected by diet (Table 4), but liver total lipid content was higher in fish fed WCO diet than in fish fed FO or ECO diets (Table 5). The higher lipid content in fish fed the WCO diet was associated with increased triacylglycerol (TAG) content (Supplementary Table 1). However, the increased liver lipid deposition was not reflected in higher HSI (Table 2).

### Fatty acid composition of muscle tissue from ECO-fed fish reflected dietary fatty acids

The fatty acid profile of muscle tissue (flesh) of fish fed the ECO diet reflected the dietary fatty acid content (Table 4). Therefore, flesh of fish fed the ECO diet had a higher EPA level than fish fed both FO and WCO diets. Similarly, the level of docosapentaenoic acid (DPA; 22:5n-3) in flesh of ECO-fed fish was similar to that found in FO-fed fish. The levels of DHA in flesh were not influenced by dietary EPA, and reflected dietary DHA contents with similar levels found between fish fed ECO and WCO diets. Total n-3 LC-PUFA (20:4n-3 + EPA + DPA + DHA) was higher in fish fed the ECO diet (23.8%) than in fish fed WCO (12.6%), but lower than in FO-fed fish (29.3%). Total saturated fatty acids in flesh of fish fed the ECO diet were intermediate between the levels in fish fed the FO and WCO diets, which were higher and lower, respectively. However, monounsaturated fatty acid content was lowest in fish fed the ECO diet, and total n-6 PUFA levels of flesh were higher in fish fed both vegetable oil diets (WCO and ECO) compared to fish fed the FO diet, reflecting dietary levels.

### High DHA levels in liver of fish fed the ECO diet indicated active DHA biosynthesis

Similar to muscle tissue, the EPA level in liver of fish fed the ECO diet was higher than in fish fed either of the other diets (Table 5). However, in contrast to muscle, DPA level in liver was also highest in fish fed the ECO diet compared to fish fed either the FO or WCO diets. Furthermore, liver DHA contents differed significantly, being higher in liver of ECO-fed fish than in liver of fish fed WCO, but lower than in fish fed FO. The higher DPA and DHA levels in liver of ECO-fed fish compared to those in livers of WCO-fed fish suggested active biosynthesis and accumulation of these fatty acids in fish fed the high EPA feed. In contrast, the highest percentages of metabolites derived from 18:3n-3 (18:4n-3, 20:3n-3 or 20:4n-3) were observed in liver of fish fed WCO. There was higher n-6 PUFA in livers of fish fed both the vegetable oil diets compared to fish fed FO with 18:2n-6 being highest in fish fed WCO and ARA being highest in fish fed ECO. In absolute terms (mg of fatty acid per 100 g of tissue), EPA and DPA content were higher in ECO-fed fish than in fish fed the other dietary treatment. Overall, in absolute terms, liver of fish fed the ECO diet showed no significant difference in total n-3 PUFA content compared to fish fed FO (Supplementary Table 1).

### The vegetable oil diets induced a similar number of differentially expressed genes compared to FO

**Figure 1:**
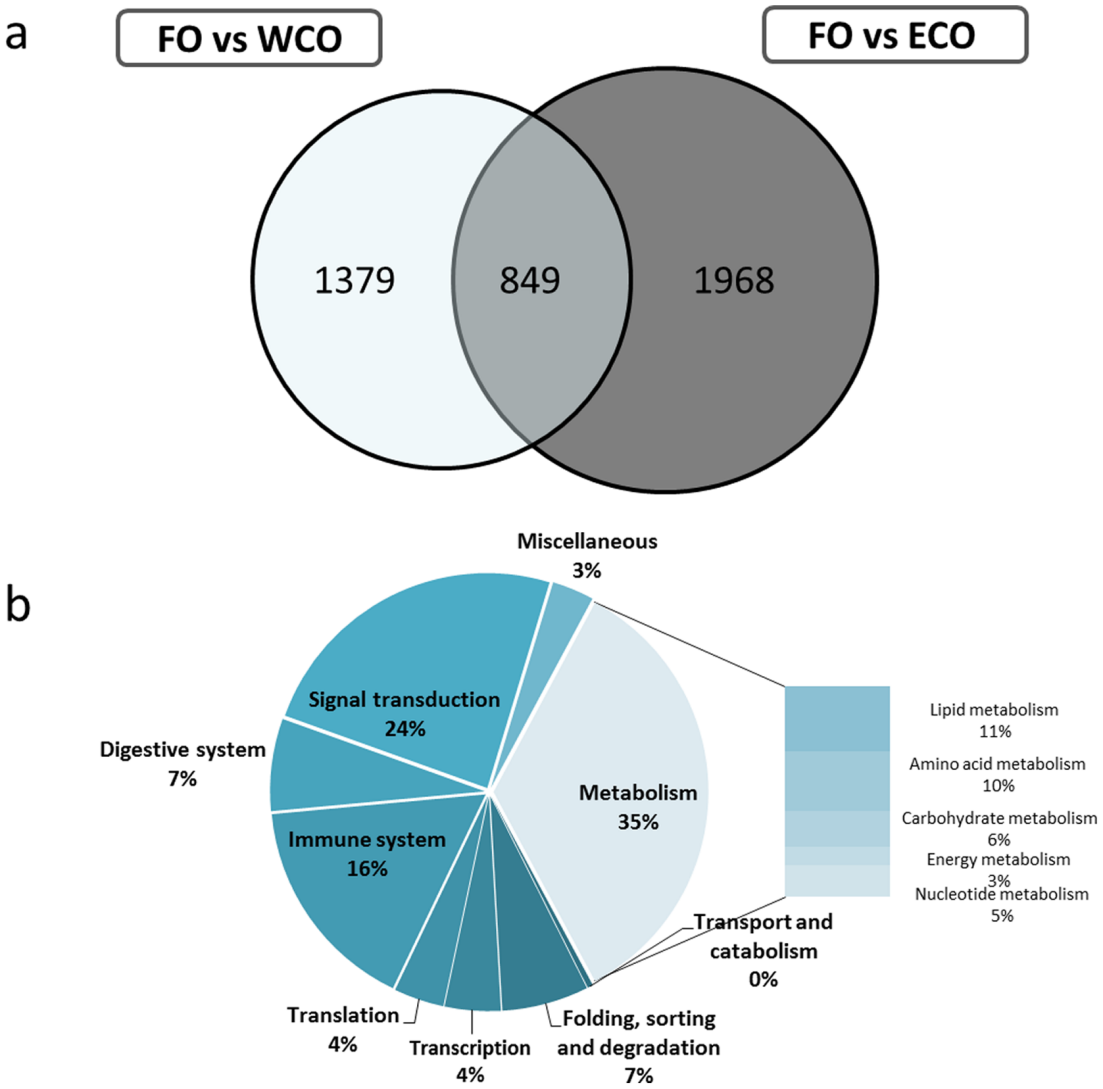
Impact of diet on liver transcriptome of Atlantic salmon given feeds containing Camelina oils (ECO and WCO) in comparison with fish fed fish oil (FO). (A) Venn diagram representing mRNA transcripts differentially expressed in the liver of Atlantic salmon fed the experimental diets WCO and ECO compared to diet FO. The area of the circles is scaled to the number of transcripts (Welch t-test, p < 0.05). (B) Distribution by categories of common differentially expressed genes (428) in liver between Atlantic salmon fed WCO and ECO when compared to FO-fed fish (Welch t-test, p < 0.05). Non-annotated genes and features corresponding to the same gene are not represented.

Microarray analysis of liver transcriptomes returned 2228 and 2817 differentially expressed gene (DEG) features when FO-fed fish were compared to WCO- and ECO-fed fish, respectively, with 849 probes common between both contrasts (Fig. 1A). The common DEG were subjected to more detailed analysis by assigning them KO numbers and mapping them to a known compendium of metabolic pathways (KEGG). The pathways commonly affected in both contrasts were those related to cell communication (68% of genes in common), circulatory system (67%), xenobiotic degradation and metabolism (61%) and lipid metabolism (56%) (Fig. 1B). When the list was reduced to the top 100 differentially expressed features, the main category affected was metabolism (70.4%), particularly lipid (59.3%) followed by carbohydrate (5.6%) metabolism (Supplementary Table 2). The high representation of lipid metabolism was partly due to some genes being represented by several features, particularly those related to fatty acid desaturation (*delta-6* and *5 fatty acyl desaturase*) and cholesterol metabolism (*isopentenyl-diphosphate delta isomerase*).

### The metabolic response in liver of fish fed ECO was more similar to that in fish fed WCO compared to fish fed FO

**Figure 2:**
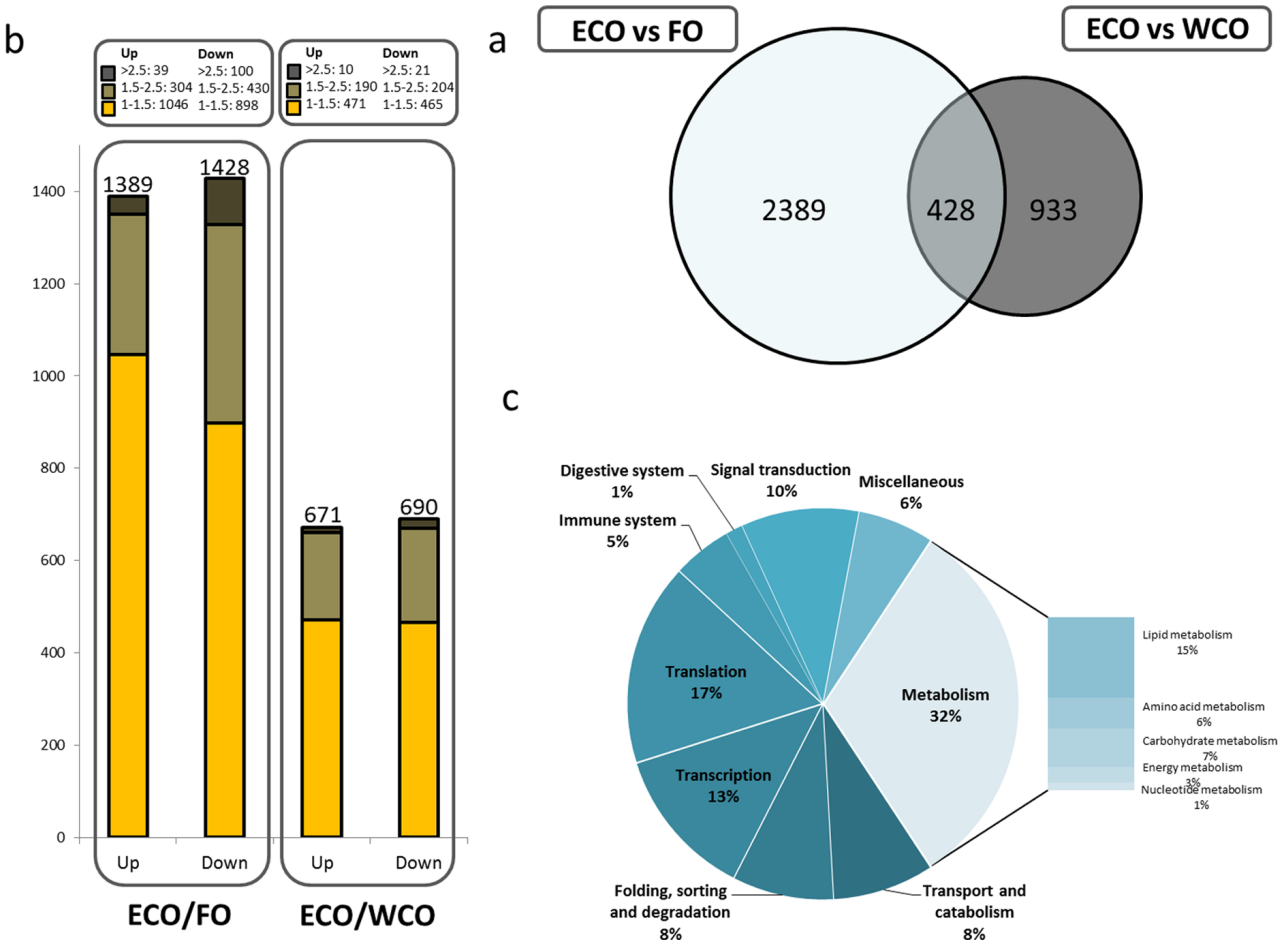
Impact of diet on liver transcriptome of Atlantic salmon given a feed containing oil from transgenic Camelina (ECO) in comparison with fish given feeds containing fish oil (FO) or wild-type Camelina oil (WCO). (A) Venn diagram representing mRNA transcripts differentially expressed in the liver of Atlantic salmon fed the ECO diet compared to fish fed the WCO and FO diets. The area of the circles is scaled to the number of transcripts (Welch t-test, p < 0.05). (B) Numbers of DEG ranked by fold-change and direction of change, up- or down, in fish fed ECO compared to fish fed FO or WCO (C) Distribution by categories of common differentially expressed genes (428) in liver between Atlantic salmon fed ECO compared to fish fed FO and WCO (Welch t-test, p < 0.05). Non-annotated genes and features corresponding to the same gene are not represented.

Statistical analysis of the microarray data returned a list of 2817 and 1361 DEG in liver of salmon fed ECO were compared to fish fed FO or WCO respectively, (p < 0.05; Fig. 2A). From these transcripts, 2389 were exclusive to the ECO v. FO comparison (p < 0.05), of which 747 were regulated at a fold change (FC) > 1.5 (31.3% of the total number of transcripts) (Fig. 2B). When comparing liver from salmon fed ECO with WCO, 933 probes exclusive to this contrast were identified, of which 319 had a FC > 1.5 (34.2%) (Figs. 2A and 2B). A total of 428 transcripts were ECO specific (common to both contrasts, Fig. 2A) and, of these, only 9 showed expression changes in opposite directions (ECO/FO and ECO/WCO), indicating that the transcriptomic response was consistent. After removing non-annotated genes, KEGG analysis of the common 428 transcripts returned 148 KO terms at p < 0.05 and revealed that the most affected biological categories were metabolism (32%), translation (17%) and transcription (13%) (Fig. 2C). More comprehensive analysis of the top 100 most significant hits and fold differences showed increased representation of metabolism, and particularly lipid metabolism (Supplementary Table 3). The differing distribution was mainly due to the repetition of multiple features of *isopentenyl-diphosphate delta isomerase* (*idi*) gene and to the lower significances found in the other categories. The high fold-changes (FC) found in the lipid metabolism category were noteworthy, especially when comparing ECO-fed fish to to FO-fed fish, with an average FC of −4.14 for *idi*, −4.69 for *farnesyl pyrophosphate synthetase* (*fdps*) or −4.95 for *squalene epoxidase* (*sqle*), all genes involved in cholesterol/isoprenoid biosynthesis. Carbohydrate metabolism category was also augmented and it is remarkable the high FC (−6.18) in the ECO/FO contrast of the *MGC80816 protein*, involved in butanoate metabolism. Other categories affected were translation (16.7%), transport (6.3%), protein folding (4.2%), signalling (4.2%), transcription (2.1%) and digestive system (2.1%).

### The microarray analyses data were fully validated by qPCR of selected genes

Validation of the microarray data was performed by comparing the expression of 10 genes, selected according to their significant up- or down-regulation including some belonging to the LC-PUFA biosynthesis pathway that were identified by KEGG analysis as being significantly affected by diet (Table 6). Good correspondence in terms of intensity (FC) and direction of change (up- or down-regulation) was observed among the studied genes (90%, 9 out of 10). However, the match was less consistent in terms of significance (p value) when comparing microarray and qPCR results which could be due to the relatively small changes in expression observed in all the genes for both analyses.

### No transgenic EPA-Camelina DNA fragments could be detected in liver, pyloric caeca or muscle

**Figure 3:**
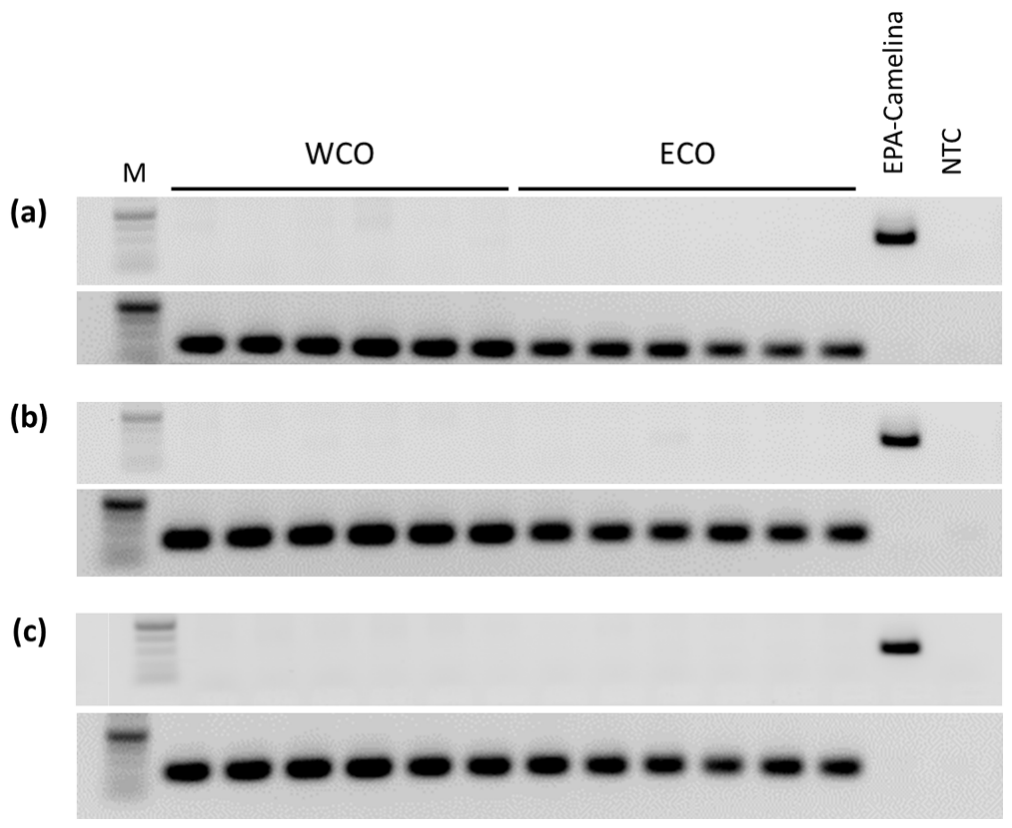
Cropped gel showing PCR products from liver (a), pyloric caeca (b) and muscle/flesh (c) of Atlantic salmon fed either WCO or ECO diets for *nptII* (transgenesis marker; top image) or *gh* (fish gene, bottom image). All the gels were run under the same experimental conditions. Lanes 1–6, Atlantic salmon fed WCO diet; Lanes 7–12, Atlantic salmon fed ECO diet; EPA-Camelina, transgenic Camelina seed cake; M, marker; NTC, non template control.

All salmon tissues tested negative for the presence of the Camelina T-DNA gene construct, as monitored by the use of *nptII* primers, directed towards the transgene construct sequence, whereas all tissues tested positive to salmon growth hormone gene, *gh* (Fig. 3).

## Discussion

Inclusion of the EPA-rich Camelina oil in diets for juvenile Atlantic salmon had no detrimental effects on fish growth or performance after seven weeks of feeding the experimental diets. Furthermore, performance did not differ between fish fed any of the three diets, confirming that the oil from transgenic Camelina could be employed as a replacement for fish oil in feeds for Atlantic salmon. The fish performance data obtained were consistent with those reported previously in other feeding trials where fish oil was substituted by equivalent wild-type Camelina oil in juvenile Atlantic cod (*Gadus morhua*)^32^ and Atlantic salmon^33^. In contrast, cod fed a diet formulated with defatted fishmeal and regular (wild-type) Camelina oil displayed lower weight gain than fish fed non-defatted fishmeal together with regular CO due to the requirements for essential fatty acids (n-3 LC-PUFA) not being met by the former diet^34^. It should be noted, therefore, that in this present trial, growth performance was not impacted in WCO-fed fish because the feeds were formulated with non-defatted fishmeal that contained sufficient n-3 LC-PUFA to satisfy salmon essential fatty acids (EFA) requirements^17^. This was as planned, as the present trial was specifically designed to investigate the effectiveness of the oil from transgenic Camelina (Tr-CO) in supplying high levels of n-3 LC-PUFA (in this case EPA), well in excess of EFA requirement levels, so that the dietary n-3 LC-PUFA would be largely deposited and stored by the fish.

Therefore, it is important to stress that n-3 LC-PUFA in fish feeds can be defined at three levels^17^. Firstly, the minimum level required to satisfy EFA requirements and thus prevent deficiency signs^35^. This level is low (<1% of diet for salmonids species) and relatively easy to supply even with today's current high demand for fish oil^16^. The second level is that required to sustain maximum growth and optimum health in fish being fed modern high-energy diets^17^. It is largely unknown in most fish species and, although higher than the minimum level, it is still relatively low. The third level, and the subject of the present study, is the level we must provide to ensure that the nutritional quality of farmed fish is maintained such that it contains n-3 LC-PUFA at similar or higher levels than can be found in wild fish. This dietary level far exceeds the biological requirements of the fish itself and cannot currently be met without the use of high levels of marine fish oil and fishmeal in the feeds, but ultimately reflects the expectations of consumer and their perception of fish such as salmon as health-beneficial.

In addition to its effectiveness as a substitute for dietary fish oil, the Tr-CO represented a high-EPA/zero DHA oil that has been hitherto unavailable and so presents unique oil with which to study n-3 LC-PUFA metabolism, including EPA to DHA pathways, in vertebrates. In this respect, several interesting results were obtained using this EPA-only oil. The utilisation of dietary vegetable oils has been associated with increased lipid deposition in fish tissues^32^,^33^,^36^,^37^. Results from the present study supported this, as whole fish and liver lipid content was higher in fish fed both ECO and WCO than in fish fed FO. The reduced dietary n-3 LC-PUFA, that are known to suppress TAG accumulation in mammalian pre-adipocytes^38^ or lipid accumulation in Atlantic salmon adipocytes^39^, may be the mechanism for the increased lipid deposition in fish fed vegetable oils. Indeed, fish fed WCO showed increased levels of TAG, which is in agreement with previous studies^32^,^34^. However, fish fed the ECO diet had similar liver n-3 PUFA levels (other than DHA) as FO-fed fish, and showed increased hepatic lipid levels, which could indicate that the factor determining the increased adiposity could be reduced DHA levels, rather than overall n-3 PUFA levels.

The ECO diet had differing effects on fatty acid deposition depending on tissue. As expected of a tissue that serves as a lipid/energy store in salmon, muscle largely reflected dietary fatty acid composition^15^. Higher levels of desaturation products of 18:3n-3 and 18:2n-6 observed in muscle of ECO-fed fish compared to WCO-fed fish probably reflected the higher dietary content of these fatty acids rather than active biosynthesis. Nonetheless, limited activation of the LC-PUFA biosynthesis pathway was observed in fish fed WCO as shown by increased 18:4n-3 compared to fish fed FO. Increased elongation products of 18:3n-3 and 18:2n-6, 20:3n-3 and 20:2n-6, observed in fish fed both ECO and WCO compared to fish fed FO were consistent with data from many studies that report an increase in these elongation products after feeding vegetable oils in fish species^32^,^40^,^41^,^42^. Inhibition of this elongation may be another example of a DHA effect that dietary EPA alone cannot replicate. Importantly though, muscle DHA did not vary between fish fed the ECO or WCO diets and, although its level was higher than that found in those diets, this was likely due to selective retention of DHA reported previously in many studies where salmon were fed dietary vegetable oil^33^,^43^,^44^. Most importantly, from a human health viewpoint, the ECO-fed salmon would have more beneficial effects compared to WCO-fed fish due to total n-3 LC-PUFA content in muscle being nearly twice that in fish fed WCO, albeit not as high as in fish fed FO.

In contrast to muscle, liver exhibited active LC-PUFA biosynthesis in fish fed the ECO diet, with higher DHA levels than those found in fish fed WCO. These results were supported by up - regulation of *fads2d6*, *fads2d5* and *elovl2* gene expression in the liver of fish fed ECO, as expected for the operation of the LC-PUFA biosynthesis pathway in salmonids including the Sprecher shunt^45^. These genes were up-regulated in Atlantic salmon juveniles fed a vegetable oil enriched with EPA, whereas, the inclusion of EPA plus DHA inhibited their expression^46^. The results in the present study indicate that the relative lack of dietary DHA in diets ECO and WCO induced expression of the enzymes involved in its biosynthesis, and that high dietary EPA (as in ECO) did not reduce their expression. Interestingly, production of shorter chain products derived from 18:3n-3, such as 18:4n-3, 20:3n-3 and 20:4n-3, were not observed in the liver of ECO-fed fish. In contrast, WCO-fed fish that also displayed up-regulated expression of *fads2d6*, *fads2d5* and *elovl2*, show increased levels of 18:4n-3, 20:3n-3 and 20:4n-3. The lower level of these intermediates in fish fed ECO is likely due to the high concentration of EPA found in liver, which would compete with 18:3n-3 at the level of *fads2d6*.

Dietary impacts on metabolism were determined by investigating the transcriptome of liver as the main organ integrating, processing and distributing dietary lipid^47^. Several studies analysing fish transcriptomic responses after substitution of dietary fish oil with vegetable oils have shown that, regardless of oil employed, metabolism and in particular lipid metabolism, are highly affected categories^47^,^48^,^49^,^50^,^51^ For instance, genes involved in LC-PUFA biosynthesis were overrepresented among the DEG in liver of Atlantic salmon fed vegetable oils^47^. In the present study, this applies to fish fed both WCO and ECO diets, where these genes occupy the top positions among the 100 highest expressed genes in terms of intensity, when compared to fish fed FO. Another metabolic pathway significantly affected by dietary vegetable oil was sterol metabolism. Vegetable oils are both low in cholesterol and can also be rich in phytosterols, which have been shown to reduce plasma cholesterol, LDL-cholesterol and LDL-TAG in brook trout (*Salvelinus fontinalis*)^52^ and LDL-cholesterol in humans by inhibiting intestinal cholesterol intake^53^. It was also evident that the ECO feed affected pathways related to carbohydrate metabolism, which represented 10% of the common DEG between the ECO/FO and ECO/WCO contrasts. Alteration of this category is not surprising, as pathways of lipogenesis, lipolysis, glycolysis, gluconeogenesis and pentose phosphate shunt are interrelated in Atlantic salmon^48^ and thus, alterations in lipid metabolism may also affect carbohydrate metabolism.

In conclusion, genetically engineered Camelina has proved to be a feasible source of n-3 LC-PUFA which could effectively substitute for FO in aquafeeds and provide levels of these important fatty acids in farmed fish that could maintain their nutritional quality for the human consumer. The oil investigated in the present study, characterised by the presence of 20% EPA, was entirely suitable for feeding Atlantic salmon. It maintained the nutritional quality of the fillet, as high levels of EPA and also DPA, both highly beneficial for human health, were accumulated. Furthermore, increased levels of DPA and DHA were accumulated in the liver, accompanied by an increase in the expression of the enzymes involved in the endogenous biosynthesis of LC-PUFA. Moreover, this high-EPA/zero DHA oil triggered a metabolic response similar to that generated by wild-type Camelina oil, mainly affecting lipid metabolism pathways, which indicated that a combination of EPA and DHA confers the metabolic properties of FO rather than EPA alone.

## Methods

### Metabolic engineering of Camelina

A construct containing a cassette of five genes was used for transformation^30^. Briefly, the five-gene construct contained a set of genes optimised for EPA synthesis: a Δ6-desaturase gene from *Ostreococcus tauri* (OtΔ6), a Δ6 fatty acid elongase gene from *Physcomitrella patens* (PSE1), a Δ5-desaturase gene from *Thraustochytrium* sp. (TcΔ5), a Δ12-desaturase gene from *Phytophthora sojae* (PsΔ12) and an ω3-desaturase from *Phytophthora infestans* (Pi- ω3) as described in detail previously^30^. All genes were individually cloned under the control of seed-specific promoters, and then combined into a single T-DNA transformation vector as described previously^28^. The destination vector contained an NPTII gene with the nos promoter as a selection marker. All open reading frames for desaturases and elongases were re-synthesised and codon-optimised for expression in *C. sativa*.

### Production of Camelina oil

*C. sativa* was grown in a controlled-environment chamber at 23°C day/18°C night, 50–60% humidity, and kept under a 16 h photoperiod (long day) at 250 μmol m^−2^ sec^−1^. Oil (7 kg) was produced from seeds (approximately 20 kg) by cold-pressing and solvent extraction to maximise yield (~35%) (PPM, Magdeburg, Germany). The anti-oxidant ethoxyquin was added to stabilise the final product.

### Diets and feeding trial

Three isonitrogenous and isoenergetic diets were formulated to satisfy the nutritional requirements of salmonid fish (Supplementary Table 4)^17^. The diets supplied 46 g.kg^−1^ crude protein and 21 g.kg^−1^ crude lipid at a crude energy level of 22.5 MJ.kg^−1^ and were manufactured at BioMar Tech-Centre (Brande, Denmark). The three feeds were produced by vacuum coating identical dry basal extruded pellets with either fish oil (FO), wild-type Camelina oil (WCO) or EPA-Camelina oil (ECO) (Supplementary Table 4). Non-defatted fishmeal was employed as the major protein source to ensure EFA requirements were met^17^. A total of 405 juvenile (post-smolt) Atlantic salmon with an average body weight of 82.5 ± 8.1 g (mean ± S.D.) were distributed between 9 seawater tanks (45 per tank) and fed one of the three experimental feeds in triplicate for 7 weeks. Prior to the start of the experimental period fish were fed a standard commercial feed containing 30% fish oil and 70% rapeseed oil. The experimental system comprised 1 m^2^, 500 L tanks supplied by flow-through seawater (15 L min^−1^) at ambient temperature that averaged 10.2 ± 0.6°C. Experimental feeds were delivered in excess by automatic disc feeders with an automated uneaten feed collection system in order to determine accurate feed efficiency. At the end of the trial fish were weighed and growth rate, feed efficiency and biometric parameters calculated as follows: Specific growth rate (SGR) = 100 * (lnWf – lnWo)/t, where Wo = initial weight (g) and Wf = final weight (g) at time t (days). Feed conversion ratio (FCR) = FI/(Bf - Bo), where FI = feed intake (g), Bo = initial biomass (g) and Bf = final biomass (g). Fulton's condition factor (k) = 100 * (W/L^3^), where W is the final weight (g) and L is the total length (cm). Liver and empty gastrointestinal tract were used to calculate hepatosomatic index (HSI) = liver weight (g) * 100/Wf (g) and viscerosomatic index (VSI) = liver + gastrointestinal tract (g) * 100/Wf (g).

### Sample collection

At the end of the trial, fish were not fed for 48 h prior to being anaesthetised and killed by overdose with metacaine sulphonate (MS222). Three whole fish per tank (9 per treatment) were frozen for analyses of proximate composition. A further 9 fish per tank were used for biometric measurements (hepato-somatic and viscera-somatic indices) and tissue analyses. Samples of flesh (Norwegian quality cut; NQC) and liver from 3 fish per tank were immediately frozen in liquid nitrogen and stored at −70°C prior to total lipid and fatty acid analyses. Further samples of liver were collected from six fish per treatment (two per tank) and stabilised in RNAlater® (Sigma, Poole, UK) prior to RNA extraction.

### Proximate composition

Diets and whole fish were ground before determination of proximate composition according to standard procedures^54^. Fish were pooled per tank and three technical replicates for single batch diet were analysed. Moisture contents were obtained after drying in an oven at 110°C for 24 h and ash content determined after incineration at 600°C for 16 h. Crude protein content was measured by determining nitrogen content (N × 6.25) using automated Kjeldahl analysis (Tecator Kjeltec Auto 1030 analyzer, Foss, Warrington, UK) and crude lipid content determined gravimetrically after Soxhlet lipid extraction (Tecator Soxtec system 2050 Auto Extraction apparatus).

### Lipid class and fatty acid compositions

Samples of muscle (flesh) and liver from three fish per tank were prepared as pooled homogenates and total lipid extracted from 1 g by homogenising in chloroform/methanol (2:1, v/v) using an Ultra-Turrax tissue disrupter (Fisher Scientific, Loughborough, UK), and content determined gravimetrically^55^. Lipid classes were separated by double-development, high-performance thin-layer chromatography (HPTLC)^56^. Total lipid samples (1–2 μg) were applied and the plates developed in methyl acetate/isopropanol/chloroform/methanol/0.25% aqueous KCl (25:25:25:10:9, by vol.). Excess solvent was evaporated via air drying and vacuum desiccation and plates developed to 9.5 cm using a solvent mixture containing isohexane/diethyl ether/acetic acid (80:20:1, by vol.). Lipid classes were visualised by spraying with 3% (w/v) aqueous cupric acetate containing 8% (v/v) phosphoric acid and charring plates at 160°C for 20 min. Lipid classes were quantified by densitometry using a CAMAG-3 TLC scanner (version Firmware 1.14.16; CAMAG, Muttenz, Switzerland). Fatty acid methyl esters (FAME) were prepared from total lipid by acid-catalysed transesterification at 50°C for 16 h^57^, using heptadecanoic acid (17:0) as an internal standard. FAME were extracted and purified as described previously^57^. FAME were separated and quantified by gas-liquid chromatography using a Fisons GC-8160 (Thermo Scientific, Milan, Italy) equipped with a 30 m × 0.32 mm i.d. × 0.25 μm ZB-wax column (Phenomenex, Cheshire, UK), on-column injector and a flame ionization detector. Data were collected and processed using Chromcard for Windows (version 2.01; Thermoquest Italia S.p.A., Milan, Italy). Individual FAME were identified by comparison to known standards and published data^58^.

### RNA extraction

Liver from six individual fish per dietary treatment were homogenised in 1 ml of TriReagent® (Sigma-Aldrich, Dorset, UK) RNA extraction buffer using a bead tissue disruptor (Bio Spec, Bartlesville, Oklahoma, USA). Total RNA was isolated following manufacturer's instructions and quantity and quality determined by spectrophotometry using a Nanodrop ND-1000 (Labtech Int., East Sussex, UK) and electrophoresis using 500 ng of total RNA in a 1% agarose gel.

### Microarray hybridisations and image analysis

Transcriptome analysis was performed in liver tissues using an Atlantic salmon custom-made oligoarray with 44 k features per array on a four-array-per-slide format (Agilent Technologies UK Ltd., Wokingham, UK). The probes were co-designed by researchers at the Institute of Aquaculture (University of Stirling, UK) and the Norwegian Institute of Food, Fisheries and Aquaculture Research (Nofima, Tromsø, Norway). A dual-label experimental design was employed for the microarray hybridisations with Cy3-labelled test samples competitively hybridised to a common Cy5-labelled pooled-reference per array. A total of 36 arrays were utilised, one array per individual fish and tissue. The common reference was a pool of equal amounts of amplified RNA from all test samples.

Indirect labelling methodology was employed in preparing the microarray targets. Amplified antisense RNA (aRNA) was produced from each RNA sample using TargetAmpTM 1-Round Aminoallyl-aRNA Amplification Kit 101 (Epicentre, Madison, Wisconsin, USA), as per manufacturer's methodology, followed by Cy3 or Cy5 fluor incorporation through a dye-coupling reaction. Microarray hybridisations were performed in SureHyb hybridisation chambers in a DNA Microarray Hybridisation Oven (Agilent Technologies). For each hybridisation, 825 ng of Cy3-labelled experimental biological replicate and Cy5-labelled reference pool were combined and total volume made up to 35 μl with nuclease-free water. Detailed information regarding the microarray hybridisations and image analysis has been published previously^48^.

### Quantitative real time PCR

Expression of candidate genes of interest (fatty acyl desaturases 5 and 6 and fatty acyl elongases 2, 5a and 5b) was determined by quantitative PCR (qPCR) in liver from fish fed all diets, as well as genes for microarray validation (Supplementary Table 5). Results were normalised using reference genes, elongation factor 1-α and cofilin-2, which were chosen as the most stable according to GeNorm. cDNA was synthesised using 2 μg of total RNA and random primers in 20 μl reactions and the High capacity reverse transcription kit without RNase inhibiter according to the manufacturer's protocol (Applied Biosystems, Warrington, UK). The resulting cDNA was diluted 20-fold with milliQ water. The efficiency of the primers for each gene was previously evaluated to ensure that it was close to 100%. qPCR was performed using a Biometra TOptical Thermocycler (Analytik Jena, Goettingen, Germany) in 96-well plates in duplicate 20 μl reaction volumes containing 10 μl of SYBR Green RT-PCR Master Mix (Applied Biosystems), 1 μl of the primer corresponding to the analyzed gene (10 pmol), 3 μl of molecular biology grade water and 5 μl of cDNA, with the exception of the reference genes, which were determined using 2 μl of cDNA. In addition amplifications were carried out with a systematic negative control (NTC-non template control) containing no cDNA. Standard amplification parameters contained an initial activation step at 95°C for 15 min, followed by 35 cycles: 15 s at 95°C, 30 s at the annealing Tm and 30 s at 72°C.

### Tracking of the nptII gene in Atlantic salmon liver, pyloric caeca and muscle

Genomic DNA was extracted from fish flesh, pyloric caeca and liver using REALPURE extraction kit (Valencia, Spain) according to the manufacturer's instructions. Briefly, tissue samples were incubated in 300 μl of lysis solution overnight at 55°C with 3 μl of Proteinase K. Following the incubation, samples were cooled down and RNase treatment performed (37°C for 60 min). After protein precipitation, DNA was precipitated by adding 300 μl of isopropanol and hydrated with 5 mM Tris. Total DNA was quantified by spectrophotometry and quality determined by electrophoresis as described above. Two primers pairs targeting an endogenous Atlantic salmon gene (growth hormone) and a transgene marker (Kanamycin resistance gene, nptII) were used (Supplementary Table 5). Fifty ng of extracted DNA was used in PCR amplifications which were performed in a final volume of 10 μl, containing 5 μl of MyTaq™ HS Mix (Bioline, London, UK). Each set of PCR included a positive control (DNA from EPA-Camelina) and a non-template control (NTC).

### Statistical analysis

All data are means ± S.E. (n = 3) unless otherwise specified. Percentage data for survival, proximate composition, lipid content and fatty acid composition were subjected to arcsin square-root transformation prior to statistical analyses. Data were tested for normality and homogeneity of variances with Levene's test prior to one-way analysis of variance followed by a Tukey-Kramer HSD multiple comparisons of means. All statistical analyses were performed using SPSS software (IBM SPSS Statistics 19; SPSS Inc., Chicago, IL, USA). Statistical analysis of microarray hybridization data was performed in GeneSpring GX version 12.6.1 (Agilent Technologies, Wokingham, Berkshire, UK) using a Welch (unpaired unequal variance) t-test, at 0.05 significance. No multiple test correction was employed as previous analyses indicated that they were over-conservative for these nutritional data^48^,^49^. Data were submitted to the Kyoto Encyclopedia of Genes and Genomes (KEGG) for biological function analysis. Gene expression results were analyzed using the relative expression software tool (REST 2009), which employs a pairwise fixed reallocation randomization test (10,000 randomizations) with efficiency correction^59^ to determine the statistical significance of expression ratios (gene expression fold changes) between two treatments.

## Author Contributions

M.B.B. performed, interpreted and evaluated all biological, molecular and bioinformatic analysis. M.S. performed the biochemical analyses. Gene constructs were designed by J.A.N. and O.S. with vector construction and plant transgenesis performed by O.S. All agronomy, seed harvest and analyses was performed by S.U. The salmon trial was designed by P.J.C., D.R.T. and M.B.B. and supervised by P.J.C., D.R.T. and J.A.N. were responsible for the study design and supervised the entire work. The manuscript was written by D.R.T. and M.B.B. All authors discussed and commented on the manuscript.

## Supplementary Material

Supplementary Information Supplementary Information

## Figures and Tables

**Table 1:** Fatty acid composition (percentage of fatty acids) of the oils and feeds

	Oils			Feeds		
	Fish	Wt-CO	Tr-CO	FO	WCO	ECO
14:0	7.50	0.06	n.d.	7.20	1.16	1.30
16:0	17.98	5.14	6.65	18.96	7.79	9.03
18:0	3.56	2.63	5.57	3.72	2.70	4.64
20:0	0.22	1.58	2.37	0.21	1.22	2.06
**Σ saturated**^1^	**29.91**	**9.86**	**15.54**	**30.69**	**13.20**	**17.38**
16:1n-7	8.90	0.12	1.78	7.96	1.30	1.25
18:1n-9	7.70	17.35	6.36	9.84	16.10	6.10
18:1n-7	3.07	1.09	0.00	3.30	2.24	1.85
20:1n-9	1.40	14.69	7.78	2.47	12.72	6.68
22:1n-11	1.02	2.55	0.96	2.34	3.45	1.84
**Σ monounsaturated**^2^	**22.77**	**36.39**	**16.88**	**26.58**	**36.42**	**18.22**
18:2n-6	1.21	19.26	19.80	3.60	17.23	19.12
18:3n-6	0.31	0.00	1.77	0.25	0.05	1.61
20:2n-6	0.14	1.53	1.84	0.16	1.23	1.21
20:3n-6	0.20	0.00	1.19	0.07	0.00	1.19
20:4n-6	0.98	0.06	2.86	0.88	0.13	2.54
**Σ n-6 PUFA**^3^	**3.30**	**20.85**	**27.46**	**5.28**	**18.65**	**25.72**
18:3n-3	0.85	31.88	11.17	1.01	24.96	10.45
18:4n-3	3.18	0.00	1.00	2.45	0.44	1.40
20:3n-3	0.08	1.01	1.35	0.07	0.76	0.97
20:4n-3	0.81	0.00	3.30	0.66	0.10	3.00
20:5n-3	18.76	0.00	20.36	15.95	2.14	18.75
22:5n-3	2.15	0.00	1.42	1.81	0.27	1.02
22:6n-3	12.35	0.00	0.00	11.23	2.50	2.42
**Σ n-3 PUFA**	**38.20**	**32.89**	**38.60**	**33.19**	**31.17**	**38.03**
**Σ PUFA**^4^	**47.32**	**53.74**	**66.06**	**42.73**	**50.38**	**64.40**
**Total n-3 LC-PUFA**	**34.05**	**0.00**	**25.08**	**29.55**	**5.01**	**25.19**

Data expressed as means of three technical replicates per batch of diet.

^1^contains 15:0, 22:0 and 24:0;

^2^contains 16:1n-9, 20:1n-11, 20:1n-7, 22:1n-9 and 24:1n-9;

^3^contains 22:4n-6 and 22:5n-6;

^4^contains C16 PUFA. Fish and FO, fish oil and respective feed; LC- PUFA, long-chain polyunsaturated fatty acid (sum of 20:4n-3, 20:5n-3 22:5n-3 and 22:6n-3); n.d. not detected; Tr-CO and ECO, oil from transgenic Camelina and respective feed; Wt-CO and WCO, oil from wild-type Camelina and respective feed.

**Table 2:** Growth performance, survival, feed utilisation and basic biometry over the 7-week experimental period

	FO	WCO	ECO
Final weight (g)	196.5 ± 26.3	200.5 ± 28.5	207.9 ± 26.5
Total length (cm)	25.0 ± 1.0	24.8 ± 1.4	25.0 ± 1.0
Survival (%)	100.0 ± 0.0	100.0 ± 0.0	100.0 ± 0.0
HSI	0.9 ± 0.1	0.9 ± 0.1	0.9 ± 0.1
VSI	9.5 ± 0.9	9.6 ± 1.2	9.9 ± 0.7
FI (g/tank)	4200.0 ± 155.0	4203.0 ± 97.0	4257.0 ± 216.0
FCR	0.9 ± 0.0	0.9 ± 0.0	0.9 ± 0.0
SGR	1.9 ± 0.0	1.9 ± 0.0	2.0 ± 0.1
k	1.3 ± 0.0	1.3 ± 0.0	1.3 ± 0.0

Data are means ± SD (n = 3). There were no significant differences between treatments in any parameter. FCR, feed conversion ratio; FI, feed intake; HSI, hepato-somatic index; k, condition factor; SGR, specific growth rate; VSI, viscera-somatic index.

**Table 3:** Whole body proximate composition (%) at initiation of the dietary trial and after 7 weeks of feeding the experimental diets

	Initial	FO	WCO	ECO
Dry matter	28.4 ± 1.2	29.8 ± 0.3^b^	30.7 ± 0.4^a^	30.3 ± 0.1^ab^
Protein	17.8 ± 0.5	17.5 ± 0.0	17.5 ± 0.2	17.3 ± 0.1
Lipid	8.0 ± 0.2	9.7 ± 0.2^b^	10.6 ± 0.3^a^	10.4 ± 0.1^a^
Ash	2.2 ± 0.6	2.0 ± 0.0	1.9 ± 0.0	1.9 ± 0.1

Data are means ± SD (n = 3). Different superscript letters within a row denote significant differences among diets. Statistical differences between dietary treatment only (not initial) were determined by one-way ANOVA with Tukey's comparison test (p < 0.05).

**Table 4:** Lipid content (percentage of wet weight) and fatty acid compositions (percentage of total fatty acids) of total lipid from muscle tissue (flesh) of Atlantic salmon after 7 weeks of feeding the experimental diets

	FO	WCO	ECO
Lipid content	3.3 ± 0.3	3.9 ± 0.1	3.2 ± 0.4
14:0	5.0 ± 0.1^a^	1.6 ± 0.1^b^	1.7 ± 0.0^b^
16:0	17.3 ± 0.1^a^	11.0 ± 0.1^c^	12.1 ± 0.1^b^
18:0	3.8 ± 0.0^b^	3.4 ± 0.1^b^	4.6 ± 0.1^a^
20:0	0.2 ± 0.0^c^	0.8 ± 0.0^b^	1.1 ± 0.0^a^
**Total saturated**^1^	**26.9 ± 0.3^a^**	**17.3 ± 0.2^c^**	**20.0 ± 0.2^b^**
16:1n-7	6.1 ± 0.1^a^	1.9 ± 0.0^b^	2.0 ± 0.1^b^
18:1n-9	14.8 ± 0.3^b^	19.1 ± 0.6^a^	13.7 ± 0.6^b^
18:1n-7	3.9 ± 0.4^a^	2.4 ± 0.3^b^	2.4 ± 0.0^b^
20:1n-11	0.3 ± 0.0^a^	0.2 ± 0.0^b^	0.2 ± 0.0^ab^
20:1n-9	2.6 ± 0.1^c^	8.5 ± 0.2^a^	4.7 ± 0.0^b^
20:1n-7	0.3 ± 0.0^b^	0.3 ± 0.0^a^	0.3 ± 0.0^a^
22:1n-11	1.9 ± 0.2	1.6 ± 0.1	1.7 ± 0.1
22:1n-9	0.3 ± 0.0^c^	1.3 ± 0.0^a^	0.6 ± 0.0^b^
**Total monoenes**^2^	**31.0 ± 0.5^b^**	**36.1 ± 0.6^a^**	**26.4 ± 0.6^c^**
18:2n-6	5.2 ± 0.1^c^	13.3 ± 0.0^b^	14.5 ± 0.1^a^
18:3n-6	0.2 ± 0.0^c^	0.3 ± 0.0^b^	0.7 ± 0.0^a^
20:2n-6	0.5 ± 0.0^c^	1.4 ± 0.0^b^	1.7 ± 0.0^a^
20:3n-6	0.3 ± 0.0^c^	0.5 ± 0.0^b^	1.3 ± 0.0^a^
20:4n-6	0.9 ± 0.0^b^	0.4 ± 0.0^c^	1.7 ± 0.0^a^
**Total n-6 PUFA**^3^	**7.5 ± 0.2^c^**	**16.1 ± 0.1^b^**	**20.2 ± 0.0^a^**
18:3n-3	1.8 ± 0.0^c^	14.6 ± 0.2^a^	7.1 ± 0.1^b^
18:4n-3	1.4 ± 0.0^b^	2.0 ± 0.1^a^	1.1 ± 0.0^b^
20:3n-3	0.2 ± 0.0^b^	1.0 ± 0.0^a^	0.9 ± 0.0^a^
20:4n-3	1.1 ± 0.1^b^	1.1 ± 0.0^b^	2.4 ± 0.0^a^
20:5n-3	9.3 ± 0.3^b^	2.9 ± 0.1^c^	10.2 ± 0.4^a^
22:5n-3	3.1 ± 0.1^a^	0.9 ± 0.0^b^	3.0 ± 0.1^a^
22:6n-3	15.8 ± 0.9^a^	7.7 ± 0.5^b^	8.2 ± 0.1^b^
**Total n-3 PUFA**	**32.7 ± 0.9^a^**	**30.2 ± 0.8^b^**	**33.0 ± 0.6^a^**
**Total PUFA**^4^	42.2 ± 0.7^c^	46.6 ± 0.8^b^	53.6 ± 0.6^a^
**Total n-3 LC-PUFA**	**29.3 ± 0.9^a^**	**12.6 ± 0.7^c^**	**23.8 ± 0.6^b^**

Data expressed as means ± SD (n = 3). Different superscript letters within a row denote significant differences among diets. Statistical differences were determined by one-way ANOVA with Tukey's comparison test (p < 0.05).

^1^contains 15:0, 22:0 and 24:0;

^2^contains 16:1n-9 and 24:1n-9;

^3^contains 22:4n-6 and 22:5n-6;

^4^contains C16 PUFA. ECO, feed containing oil from transgenic Camelina; FO, fish oil feed; LC-PUFA, long-chain polyunsaturated fatty acids (sum of 20:4n-3, 20:5n-3, 22:5n-3 and 22:6n-3); WCO, feed containing oil from wild-type Camelina.

**Table 5:** Lipid content (percentage of wet weight) and fatty acid compositions (percentage of total fatty acids) of total lipid from liver of Atlantic salmon after 7 weeks of feeding the experimental diets

	FO	WCO	ECO
Lipid content	3.6 ± 0.1^c^	5.9 ± 0.8^a^	4.2 ± 0.2^b^
14:0	2.1 ± 0.1^a^	1.3 ± 0.1^b^	1.1 ± 0.1^b^
16:0	18.7 ± 0.9^a^	11.0 ± 1.7^c^	14.1 ± 0.8^b^
18:0	4.9 ± 0.4	4.8 ± 0.1	5.7 ± 0.1
20:0	0.1 ± 0.0^c^	0.4 ± 0.0^b^	0.5 ± 0.0^a^
**Total saturated**^1^	**26.3 ±0.5^a^**	**17.8 ± 1.6^c^**	**21.6 ± 0.9^b^**
16:1n-7	2.7 ± 0.2^a^	1.8 ± 0.1^b^	1.5 ± 0.0^b^
18:1n-9	10.5 ± 1.1^b^	22.0 ± 2.8^a^	11.8 ± 0.5^b^
18:1n-7	2.7 ± 0.2^a^	2.2 ± 0.0^b^	1.9 ± 0.1^c^
20:1n-11	0.1 ± 0.0	0.1 ± 0.0	0.1 ± 0.0
20:1n-9	1.2 ± 0.1^b^	5.9 ± 0.9^a^	2.0 ± 0.3^b^
20:1n-7	0.2 ± 0.0^b^	0.2 ± 0.0^a^	0.2 ± 0.0^b^
22:1n-11	0.4 ± 0.1	0.5 ± 0.1	0.4 ± 0.0
22:1n-9	0.1 ± 0.0^b^	0.6 ± 0.1^a^	0.2 ± 0.0^b^
**Total monoenes**^2^	**19.3 ± 1.7^b^**	**34.4 ± 4.0^a^**	**19.2 ± 0.8^b^**
18:2n-6	2.9 ± 0.4^c^	10.8 ± 0.8^a^	8.2 ± 0.7^b^
18:3n-6	0.0 ± 0.0^c^	0.4 ± 0.0^a^	0.3 ± 0.0^b^
20:2n-6	0.5 ± 0.1^b^	1.8 ± 0.1^a^	1.6 ± 0.2^a^
20:3n-6	0.2 ± 0.1^b^	1.3 ± 0.1^a^	1.3 ± 0.0^a^
20:4n-6	3.1 ± 0.3^b^	1.1 ± 0.3^c^	4.1 ± 0.2^a^
**Total n-6 PUFA**^3^	**7.3 ± 0.4^b^**	**15.5 ± 0.5^a^**	**15.8 ± 0.7^a^**
18:3n-3	1.0 ± 0.2^c^	9.1 ± 0.8^a^	3.9 ± 0.5^b^
18:4n-3	0.3 ± 0.1^c^	1.7 ± 0.1^a^	0.6 ± 0.0^b^
20:3n-3	0.2 ± 0.0^c^	1.2 ± 0.1^a^	0.8 ± 0.1^b^
20:4n-3	0.9 ± 0.1^b^	2.1 ± 0.2^a^	1.9 ± 0.1^a^
20:5n-3	9.8 ± 0.2^b^	4.1 ± 0.7^c^	10.9 ± 0.2^a^
22:5n-3	3.3 ± 0.1^b^	1.1 ± 0.2^c^	4.3 ± 0.2^a^
22:6n-3	31.5 ± 1.9^a^	12.8 ± 2.8^c^	20.9 ± 1.6^b^
**Total n-3 PUFA**^4^	**46.9 ± 1.6^a^**	**32.2 ± 2.9^b^**	**43.2 ± 0.6^a^**
**Total PUFA**	54.4 ± 1.2^b^	47.8 ± 2.5^c^	59.1 ± 0.1^a^
**Total n-3LC-PUFA**	**45.5 ± 2.0^a^**	**20.1 ± 3.6^c^**	**38.0 ± 1.2^b^**

Data expressed as means ± SD (n = 3). Different superscript letters within a row denote significant differences among diets. Statistical differences were determined by one-way ANOVA with Tukey's comparison test (p < 0.05).

^1^contains 15:0, 22:0 and 24:0;

^2^contains 16:1n-9 and 24:1n-9;

^3^contains 22:4n-6 and 22:5n-6;

^4^contains C16 PUFA. ECO, feed containing oil from transgenic Camelina; FO, fish oil feed; LC-PUFA, long-chain polyunsaturated fatty acids (sum of 20:4n-3, 20:5n-3, 22:5n-3 and 22:6n-3); WCO, feed containing oil from wild-type Camelina.

**Table 6:** Validation of microarray results by qPCR

	Liver			
	Microarray		qPCR	
	FC	p	FC	p
*fads2d6*	+2.65	0.001	+3.07	0.001
*fads2d5*	+2.91	0.002	+3.96	0.021
*elovl2*	+1.55	0.010	+1.69	0.267
*elovl5a*	+1.51	0.014	+1.13	0.716
*elovl5b*	+1.47	0.020	+1.29	0.688
*palld*	+1.18	0.006	+1.19	0.763
*ap1m2*	+1.36	0.010	+1.28	0.817
*arsb*	−1.71	0.000	+1.11	0.516
*litaf*	+1.52	0.043	+1.04	0.404
*g3bp*	+1.84	0.040	+1.34	0.883

Data shown are the fold-changes (FC) between expression levels in fish fed diets FO and ECO and p value (assessed by REST 2009).*fads2d6*, delta-6 fatty acyl desaturase; *fads2d5*, delta-5 fatty acyl desaturase; *elovl2*, fatty acyl elongase 2; *elovl5a*, fatty acyl elongase 5 isoform a; *elovl5b*, fatty acyl elongase isoform b; *palld*, pallidin; *ap1m2*, AP-1 complex subunit mu-2; *arsb*, arysulfatase B; *litaf*, lipopolysaccharide-induced tumor necrosis factor-alpha factor homolog; *g3bp*, galectin-3-binding protein.
