# Supplementary material for: A nutritionally-enhanced oil from transgenic Camelina sativa effectively replaces fish oil as a source of eicosapentaenoic acid for fish
Source: Sci Rep. 2015 Jan 29;5:8104. doi: 10.1038/srep08104 (PMC4309969; doi:10.1038/srep08104)
Supplement: Supplementary Information [file srep08104-s1.doc]

**A nutritionally-enhanced oil from transgenic Camelina sativa effectively replaces fish oil as a source of eicosapentaenoic acid for fish**

Betancor, M.B.^1*^, Sprague, M.^1^, Usher, S.^2^, Sayanova, O.^2^, Campbell, P.J.^3^, Napier, J.A.^2^, Tocher, D.R.^1^

^1^Institute of Aquaculture, School of Natural Sciences, University of Stirling, Stirling FK9 4LA, United Kingdom

^2^Department of Biological Chemistry and Crop Protection, Rothamsted Research, Harpenden AL5 2JQ, United Kingdom

^3^Biomar Ltd., North Shore Road, Grangemouth FK3 8UL, United Kingdom

**Supplementary Table 1**. Lipid classes (percentage of total lipids) and absolute fatty acid composition (mg of fatty acid per 100 g of tissue) from liver of Atlantic salmon after 7 weeks of feeding the experimental diets.

|  | **FO** | **ECO** | **WCO** |
| --- | --- | --- | --- |
| Phosphatidylcholine | 20.6±0.5^a^ | 14.1±1.4^b^ | 17.9±0.5^a^ |
| Phosphatidylethanolamine | 11.5±0.8^a^ | 8.1±0.3^b^ | 10.2±0.5^a^ |
| Phosphatidylserine | 4.1±0.5^a^ | 2.6±0.6^b^ | 3.3±0.4^ab^ |
| Phosphatidylinositol | 4.3±0.7 | 3.1±0.7 | 3.9±0.7 |
| Sphingomyelin | 3.8±0.5a | 2.4±0.2b | 3.1±0.4ab |
| Phosphatidic acid/Phosphatidylglycerol/cardiolipin | 2.5±1.1 | 1.5±1.0 | 2.1±1.1 |
| Lysophosphatidylcholine | 1.7±0.2 | 0.9±0.5 | 1.2±0.2 |
| Pigmented material | 3.8±0.8^b^ | 4.8±0.3^ab^ | 5.4±0.6^a^ |
| Unknown neutral lipid | 2.4±1.2 | 1.5±0.4 | 2.0±0.8 |
| Total polar lipids | 54.8±1.4^a^ | 39.4±4.5^b^ | 49.4±1.8^a^ |
| Total neutral lipids | 45.2±1.4^b^ | 60.6±4.5^a^ | 50.6±1.8^b^ |
| Triacylglycerols | 5.2±1.7^b^ | 19.7±4.5^a^ | 6.8±0.1^b^ |
| Diacylglycerol | 2.4±0.3^b^ | 5.8±1.0^a^ | 4.5±0.9^a^ |
| Sterols | 14.4±0.6^a^ | 10.4±0.4^c^ | 12.7±0.2^b^ |
| Free fatty acids | 17.3±1.6^b^ | 19.5±0.6^ab^ | 21.3±0.7^a^ |
| Sterol esters | 3.4±0.3 | 3.7±0.2 | 3.2±0.6 |
| Unknown neutral lipids | 2.4±1.2^b^ | 5.8±1.0^a^ | 4.5±0.9^a^ |
|  |  |  |  |
| *Fatty acid (%)* |  |  |  |
| 14:0 | 40.6±3.2^ab^ | 48.8±13.9^a^ | 26.1±1.1^b^ |
| 15:0 | 6.5±0.5^a^ | 5.5±0.9^a^ | 4.1±0.1^b^ |
| 16:0 | 353.4±23.8 | 407.3±36.5 | 341.1±15.5 |
| 18:0 | 92.0±6.2^b^ | 182.8±43.8^a^ | 137.4±6.4^ab^ |
| 20:0 | 2.8±0.6^b^ | 15.8±5.1^a^ | 13.0±1.8^a^ |
| **Total saturated^1^** | 495.6±24.2^b^ | 662.2±98.4^a^ | 523.3±21.8^ab^ |
| 16:1n-7 | 51.1±6.3^ab^ | 70.3±22.0^a^ | 37.3±2.6^b^ |
| 18:1n-9 | 199.1±26.7^b^ | 847.4±305.4^a^ | 287.0±30.8^b^ |
| 18:1n-7 | 50.9±5.2^b^ | 82.4±21.2^a^ | 45.3±5.5^b^ |
| 20:1n-11 | 1.9±0.2^b^ | 4.5±1.8^a^ | 1.9±0.3^b^ |
| 20:1n-9 | 23.2±3.3^b^ | 229.6±86.4^a^ | 49.5±9.8^b^ |
| 20:1n-7 | 3.0±0.3^b^ | 9.3±3.0^a^ | 4.5±0.7^b^ |
| 22:1n-11 | 7.0±1.8^b^ | 19.2±6.7^a^ | 9.1±1.7^b^ |
| 22:1n-9 | 1.7±0.5^b^ | 22.2±8.7^a^ | 4.3±0.7^b^ |
| **Total monoenes^2^** | 364.4±43.6^b^ | 1324.1±461.6^a^ | 466.1±50.9^b^ |
| 18:2n-6 | 55.4±9.0^b^ | 413.0±126.7^a^ | 198.2±28.5^b^ |
| 18:3n-6 | 0.7±0.2^b^ | 13.7±3.9^a^ | 6.4±0.8^b^ |
| 20:2n-6 | 10.1±1.3^b^ | 69.4±20.6^a^ | 39.6±6.9^ab^ |
| 20:3n-6 | 4.7±1.4^c^ | 46.8±7.7^a^ | 31.7±1.9^b^ |
| 20:4n-6 | 59.0±3.9^b^ | 40.4±2.6^c^ | 98.4±4.0^a^ |
| **Total n-6 PUFA^3^** | 137.8±8.0^b^ | 586.9±155.9^a^ | 384.4±41.7^a^ |
| 18:3n-3 | 18.3±3.9^c^ | 348.0±110.0^a^ | 94.2±17.3^b^ |
| 18:4n-3 | 5.4±1.4^c^ | 64.0±15.8^a^ | 13.7±0.6^b^ |
| 20:3n-3 | 3.0±0.5^c^ | 45.8±13.3^a^ | 19.2±3.9^b^ |
| 20:4n-3 | 16.7±2.8^c^ | 79.7±15.3^a^ | 47.2±4.4^b^ |
| 20:5n-3 | 184.7±9.2^b^ | 152.3±11.7^b^ | 263.7±22.7^a^ |
| 22:5n-3 | 62.8±4.3^b^ | 39.0±3.2^c^ | 103.8±8.1^a^ |
| 22:6n-3 | 592.7±34.9^a^ | 471.8±59.3^b^ | 504.6±25.5^ab^ |
| **Total n-3 PUFA^4^** | 883.6±42.3^b^ | 1200.6±184.6^a^ | 1046.4±58.5^ab^ |
| **Total PUFA** | 1025.6±41.9^b^ | 1791.4±337.4^a^ | 1432.7±98.2^ab^ |
| **Total n-3 LC-PUFA** | 856.9±44.0^ab^ | 742.9±81.6^b^ | 919.2±40.5^a^ |

Data expressed as means ± SD (n=3). Different superscript letters within a row denote significant differences among diets. Statistical differences were determined by one-way ANOVA with Tukey’s comparison test (p<0.05). ^1^contains 22:0 and 24:0; ^2^contains 16:1n-9 and 24:1n-9; ^3^contains 22:4n-6 and 22:5n-6; ^4^contains C16 PUFA. ECO, feed containing oil from transgenic Camelina; FO, fish oil feed; LC-PUFA, long-chain polyunsaturated fatty acids (sum of 20:4n-3, 20:5n-3, 22:5n-3 and 22:6n-3); WCO, feed containing oil from wild-type Camelina.

**Supplementary Table 2**.- Transcripts corresponding to the top 100 most significant features exhibiting differential expression in post-smolt Atlantic salmon liver fed FO compared to fish fed either WCO or ECO. Annotated features (58%) are arranged by functional categories and within them by increasing p value (assessed by Welch t-test). The percentage of genes distribution it is also represented after removing features belonging to the same gene

| **KO no** | | **FO/WCO** | **FO/ECO** | **Annotation** | **Accession no** |
| --- | --- | --- | --- | --- | --- |
| *Metabolism (56.0%)* | | | | |  |
| *Lipid (41.3%)* | | |  |  |  |
| K16817 | +1.57 | | -1.20 | HRAS-like suppressor 3 | S48437830 |
| K16795 | -1.36 | | -1.38 | Platelet-activating factor hydrolase IB subunit β/γ | DW569179 |
| K01823 | -3.04 | | -4.53 | Isopentenyl-diphosphate delta isomerase | CK875291 |
| K10226 | -3.68 | | -1.24 | Delta-6 fatty acyl desaturase | AY458652 |
| K01823 | -2.67 | | -1.88 | Isopentenyl-diphosphate delta isomerase | CO471940 |
| K01823 | -2.92 | | -1.83 | Isopentenyl-diphosphate delta isomerase | CK875291 |
| K01823 | -2.59 | | -4.84 | Isopentenyl-diphosphate delta isomerase | CO471940 |
| K10226 | -3.05 | | -4.06 | Delta-6 fatty acyl desaturase | AY458652 |
| K01823 | -2.23 | | -1.39 | Isopentenyl-diphosphate delta isomerase | CO471940 |
| K10226 | -2.89 | | -2.01 | Delta-5 fatty acyl desaturase | AF478472 |
| K10226 | -3.68 | | -2.78 | Delta-6 fatty acyl desaturase | CK887422 |
| K10226 | -3.61 | | -1.67 | Delta-6 fatty acyl desaturase | CK887422 |
| K01597 | -2.20 | | -1.46 | Diphosphomevalonate decarboxylase | S35514245 |
| K01823 | -3.72 | | -1.27 | Isopentenyl-diphosphate delta isomerase | S35553677 |
| K10226 | -2.24 | | -1.47 | Delta-5 fatty acyl desaturase | AF478472 |
| K13373 | -2.33 | | -2.24 | Hydroxysteroid (17-beta) dehydrogenase 7 | S35707562 |
| K00869 | -1.42 | | -2.85 | Mevalonate kinase | TC101231 |
| K10226 | -2.93 | | -2.05 | Delta-5 fatty acyl desaturase | S18892253 |
| K01823 | -2.60 | | -1.47 | Isopentenyl-diphosphate delta isomerase | CO471940 |
| K10226 | | -2.46 | -2.07 | Delta-6 fatty acyl desaturase | CK887422 |
| K10226 | | -3.55 | -2.27 | Delta-6 fatty acyl desaturase | CK887423 |
| K07748 | | -2.31 | -1.28 | NADP(P)-dependent steroid dehydrogenase | DY722086 |
| K10226 | | -3.21 | -1.16 | Delta-6 fatty acyl desaturase | CK887422 |
| K10226 | | -2.60 | -3.34 | Delta-6 fatty acyl desaturase | AY458652 |
| K10226 | | -3.37 | -1.50 | Delta-6 fatty acyl desaturase | S18892244 |
| *K12353* | | +1.32 | +3.82 | Sphingomyelin phosphodiesterase 4 | TC152561 |
| K00227 | | -2.42 | -1.19 | Lathosterol oxidase | DW471233 |
| K05917 | | -2.85 | -2.82 | 14-alpha demethylase | DY741343 |
| K10226 | | -2.23 | -1.22 | Delta-5 fatty acyl desaturase | AF478472 |
| K03846 | | 1.28 | -1.96 | Alpha-1,2-mannosyltransferase | S34422676 |
| K00787 | | -2.89 | -1.66 | Farnesyl pyrophosphate synthetase | S30244374 |
| K10226 | | -2.63 | -2.14 | Delta-6 fatty acyl desaturase | AY458652 |
|  | |  |  |  |  |
| *Carbohydrate (5.9%)* | | | | |  |
| K01689 | | +1.56 | +1.65 | Alpha-enolase | S18869572 |
| K03479 | | -1.88 | -1.87 | Alpha 2,3-sialyltransferase | CA038345 |
| K01689 | | +1.31 | +4.24 | Alpha-enolase | S18881199 |
| *Nucleotide and aminoacids (5.9%)* | | | | |  |
| K00764 | | -1.72 | -2.67 | Amidophosphoribosyltransferase | CX257577 |
| K12305 | | -1.80 | -1.87 | Ectonucleoside triphosphate diphosphohydrolase 4 | TC100521 |
|  | |  |  |  |  |
| *Energy (2.9%)* | |  |  |  |  |
| K01079 | | -2.21 | -4.01 | Phosphoserine phosphatase | gi|209733303 |
|  | |  |  |  |  |
| *Transport and catabolism (8.8%)* | | | | |  |
| K02010 | | +1.96 | +1.54 | LPS binding protein | S35590591 |
| K04144 | | +2.15 | +2.71 | RUN and FYVE domain | S15339533 |
| K07375 | | +1.53 | +2.52 | Tubulin beta | S15306231 |
|  | | | |  |  |
| *Exosome (5.9%)* | |  |  |  | TC80949 |
| K15300 | | +1.77 | +1.30 | Syntaxin binding protein 2 | TC69492 |
| K17293 | | +1.24 | +1.63 | Tetraspanin 3 |  |
|  | | | | | |
| *Transcription factors (2.9%)* | | | | |  |
| K04153 | | -2.54 | -2.50 | Sterol regulatory element binding transcription factor 2 | TC112737 |
| K04153 | | -2.74 | -3.11 | Sterol regulatory element binding transcription factor 2 | TC112737 |
|  | |  |  |  |  |
| *Signalling (5.9%)* | |  |  |  |  |
| K10162 | | +1.31 | +1.20 | BMP and activin membrane-bound inhibitor | CN181198 |
| K05853 | | -3.63 | -2.67 | Ca^2+^ transporting ATPase | CA374900 |
|  | |  |  |  |  |
| *Translation (8.8%)* | |  |  |  |  |
| K03260 | | -1.33 | -3.62 | Eukaryotic translation initiation factor 4 gamma 1 | CA373251 |
| K01873 | | -1.90 | -1.33 | Valyl-tRNA synthetase | S30260788 |
| K02937 | | -131 | -2.62 | Ribosomal protein L7 | S30243985 |
|  | |  |  |  |  |
| *Protein folding (2.9%)* | | | | | |
| K12617 | | +1.47 | +1.28 | DNA topoisomerase 2-asscoaited protein PAT1 | S32000818 |
|  | |  |  |  |  |
| *Immune system (2.9%)* | | | | |  |
| K09447 | | +1.75 | +1.56 | Interferon regulatory factor 7 | S315321112 |
|  | |  |  |  |  |
| *Miscellaneous or unknown functions (5.9%)* | | | | |  |
| K09022 | | -1.48 | -1.24 | Ribonuclease UK114 | S35525599 |
| K13103 | | -1.39 | -2.34 | Tuftelin 1 | TC95882 |

**Supplementary Table 3**.- Transcripts corresponding to the top 100 most significant features exhibiting differential expression in post-smolt Atlantic salmon liver fed ECO compared to fish fed either FO or WCO. Annotated features (57%) are arranged by functional categories and within them by increasing p value (assessed by Welch t-test). The percentage of genes distribution it is also represented after removing features belonging to the same gene

| **KO no** | | **ECO/FO** | **ECO/WCO** | **Annotation** | **Accession no** |
| --- | --- | --- | --- | --- | --- |
| *Metabolism (54.2%)* | | | | |  |
| *Lipid (37.5%)* | | |  |  |  |
| K01823 | +3.47 | | +1.80 | Isopentenyl-diphosphate delta isomerase | CO471940 |
| K01823 | +4.06 | | +2.02 | Isopentenyl-diphosphate delta isomerase | CO471940 |
| K01823 | +4.05 | | +2.02 | Isopentenyl-diphosphate delta isomerase | CO471940 |
| K01823 | +4.30 | | +2.10 | Isopentenyl-diphosphate delta-isomerase | CO471940 |
| K00787 | +4.69 | | +1.62 | Farnesyl pyrophosphate synthetase | S30244374 |
| K01823 | +4.84 | | +1.66 | Isopentenyl-diphosphate delta isomerase | CK875291 |
| K12353 | -1.51 | | -1.14 | Sphingomyelin phosphodiesterase 4 | TC152561 |
| K01852 | +4.00 | | +1.54 | Lanosterol synthase | EG755357 |
| K07748 | +3.21 | | +1.36 | NAD(P)-dependent steroid dehydrogenase-like | DY722086 |
| K00213 | +2.52 | | +1.40 | 7-dehydrocholesterol reductase | TC99602 |
| K01047 | +1.69 | | +1.20 | Phospholipase group xiia | TC74133 |
| K01597 | +3.62 | | +1.64 | Diphosphomevalonate decarboxylase | S35514245 |
| K00511 | +4.95 | | +1.48 | Squalene epoxidase | S31977705 |
| K00222 | +2.54 | | +1.51 | Transmembrane 7 superfamily member 2 | S31963099 |
| K00222 | +3.22 | | +1.67 | Transmembrane 7 superfamily member 2 | DY694469 |
| K15728 | -1.19 | | -1.16 | Lipin 2 | TC70459 |
| K00869 | +2.05 | | +1.44 | Mevalonate kinase | TC101231 |
| K17759 | +1.38 | | +1.20 | Apolipoprotein A-I-binding protein precursor | S31979685 |
|  |  | |  |  |  |
| *Carbohydrate (10.4%)* | | | | | |
| K01641 | | +6.18 | +2.37 | MGC80816 protein | CX251889 |
| K01196 | | -1.61 | -1.21 | Amylo-1, 6-glucosidase, 4-alpha-glucanotransferase | TC72565 |
| K01641 | | +2.77 | +1.77 | Hydroxymethylglutaryl-CoA synthase 1 | AM402497 |
| K00626 | | -1.35 | -1.28 | Acetyl-CoA acetyltransferase, mitochondrial precursor | S34309253 |
| K00688 | | -1.24 | -1.14 | Glycogen phosphorylase | S18870968 |
|  | |  |  |  |  |
| *Energy (2.1%)* | |  |  |  |  |
| K02144 | | +1.14 | +1.20 | ATPase, H^+^ transporting, lysosomal 50/57kDa, V1 subunit H isof 2 | S48422500 |
|  | |  |  |  |  |
| *Aminoacid (4.2%)* | | |  |  |  |
| K11182 | | +1.61 | +1.44 | Amine oxidase copper containing 3 | S30289790 |
| K10712 | | +1.54 | +1.33 | 2-aminoethanethiol dioxygenase | S30243621 |
|  | |  |  |  |  |
| *Transport (6.3%)* | | | | |  |
| K12191 | | +1.38 | +1.28 | Charged multivesicular body protein 2a | S35591015 |
| K12393 | | +1.53 | +1.27 | AP-1 complex subunit mu-2 | S15325862 |
| K13649 | | -2.49 | -1.56 | Folate receptor alpha | S35663060 |
|  | |  |  |  |  |
| *Transcription (2.1%)* | | | | |  |
| K12893 | | +1.24 | +1.19 | Splicing factor, arginine/serine-rich 4 | CA347902 |
|  | |  |  |  |  |
| *Translation (16.7%)* | | | | |  |
| K02917 | | +1.16 | +1.10 | 60S ribosomal protein L35a | EL698665 |
| K02973 | | +1.22 | +1.15 | 40S ribosomal protein S23 | S27584036 |
| K14285 | | +1.46 | +1.37 | Nuclear transport factor 2-like export factor 2 | gi|209731609 |
| K03251 | | +1.49 | +1.28 | Eukaryotic translation initiation factor 3, subunit 7 zeta | S30295618 |
| K02927 | | +1.23 | +1.27 | Ubiquitin | EG999233 |
| K02915 | | +1.21 | +1.26 | 60S ribosomal protein L34 | BX311927 |
| K13136 | | -1.35 | -1.29 | Gem associated protein 8 | TC80806 |
| K03262 | | -1.20 | -1.25 | Eukaryotic translation initiation factor 5 | TC64414 |
|  | |  |  |  |  |
| *Protein folding (4.2%)* | | | | |  |
| K10666 | | +1.40 | +1.23 | RING finger protein 185 | DW591732 |
| K11600 | | +1.50 | +1.21 | Exosome complex exonuclease RRP41 | S31973152 |
|  | |  |  |  |  |
| *Signalling (4.2%)* | | | |  |  |
| K04137 | | +2.78 | +1.47 | Alpha-1D adrenoreceptor | TC162380 |
| K04370 | | +1.21 | +1.14 | Mitogen-activated protein kinase kinase 1-interacting protein 1 | S30260572 |
|  | |  |  |  |  |
| *Digestive (2.1%)* | | | | | |
| K14463 | | -1.45 | -1.38 | Microsomal triglyceride transfer protein | CA042356 |
|  | |  |  |  |  |
| *Miscellaneous or unknown functions (10.4%)* | | | | |  |
| K06639 | | -1.55 | -1.48 | cdc14 cell division cycle 14 homolog b | TC81893 |
| K06639 | | -1.50 | -1.35 | Dual specificity protein phosphatase CDC14A | S35697443 |
| K12751 | | +1.19 | +1.11 | Myosin light polypeptide 6 | TC64796 |
|  | | +1.20 | +1.16 | Pallidin | S35697859 |
|  | | +1.62 | +1.35 | Histamine N-methyltransferase | S31979727 |

**Supplementary Table 4.** Formulations, proximate and lipid class compositions of the

experimental diets.

|  | **FO** | **WCO** | **ECO** |
| --- | --- | --- | --- |
| *Feed ingredients (%)* |  |  |  |
| Fish meal, NA LT 70 | 24.5 | 24.5 | 24.5 |
| Fish meal, SA 68 Superprime | 24.5 | 24.5 | 24.5 |
| Soy protein concentrate (60%) | 14.4 | 14.4 | 14.4 |
| Wheat gluten | 4.92 | 4.92 | 4.92 |
| Wheat | 12.8 | 12.8 | 12.8 |
| Fish oil | 17.5 | 0 | 0 |
| Wild-type Camelina oil (Wt-CO) | 0 | 17.5 | 0 |
| EPA-Camelina oil (Tr-CO) | 0 | 0 | 17.5 |
| Monocalcium phosphate | 0.59 | 0.59 | 0.59 |
| Vitamins/Minerals | 0.79 | 0.79 | 0.79 |
| Ytrium oxide | 0.05 | 0.05 | 0.05 |
|  |  |  |  |
| *Analysed composition* |  |  |  |
| Dry matter (%) | 91.84 | 91.31 | 92.60 |
| Protein (%) | 45.38 | 46.14 | 47.09 |
| Fat (%) | 24.91 | 23.15 | 23.86 |
| Ash | 8.61 | 8.48 | 8.52 |
| Gross energy (Kj/g) | 22.49 | 22.37 | 22.69 |
|  |  |  |  |
| *Lipid Classes (% Total lipid)* |  |  |  |
| Glycolipids | 0.00 | 0.00 | 0.81 |
| Phosphatidylethanolamine | 3.60 | 4.15 | 4.65 |
| Phosphatidic acid/Cardiolipin | 0.44 | 0.00 | 0.87 |
| Phosphatidylinositol | 2.60 | 2.80 | 2.45 |
| Phosphatidylserine | 1.25 | 2.65 | 0.72 |
| Phosphatidylcholine | 4.93 | 4.98 | 6.20 |
| Sphingomyelin | 1.07 | 0.62 | 0.64 |
| Lysophosphatidylcholine | 1.18 | 1.09 | 1.05 |
| Pigmented material | 0.77 | 0.42 | 0.19 |
| ***Total Polar*** | 15.84 | 16.71 | 19.51 |
| Diacylglycerols | 3.66 | 2.42 | 3.41 |
| Sterols | 10.48 | 8.83 | 8.27 |
| Free Fatty Acids | 11.99 | 11.74 | 11.38 |
| Triacylglycerols | 57.83 | 59.87 | 57.35 |
| Wax/Steryl esters | 0.20 | 0.42 | 0.58 |
| ***Total neutrals*** | 84.16 | 83.29 | 81.00 |

**Supplementary Table 5**. Primers used for qPCR or PCR analysis

| Aim | Transcript | Primer sequence (5’→3’) | Amplicon (bp) | Ta | Accession no |
| --- | --- | --- | --- | --- | --- |
| *qPCR* | *fads2d6* | F: TCCTCTGGTGCGTACTTTGT | 163 | 59˚C | NM_001123575.2^a^ |
|  |  | R: AAATCCCGTCCAGAGTCAGG |  |  |  |
|  | *fads2d5* | F: GCCACTGGTTTGTATGGGTG | 148 | 59˚C | NM_001123542.2^a^ |
|  |  | R: TTGAGGTGTCCACTGAACCA |  |  |  |
|  | *elovl2* | F: GGTGCTGTGGTGGTACTACT | 190 | 59˚C | NM_001136553.1^a^ |
|  |  | R: ACTGTTAAGAGTCGGCCCAA |  |  |  |
|  | *elovl5a* | F: TGTTGCTTCATTGAATGGCCA | 150 | 59˚C | GU238431.1^a^ |
|  |  | R: TCCCATCTCTCCTAGCGACA |  |  |  |
|  | *elovl5b* | F: CTGTGCAGTCATTTGGCCAT | 192 | 59˚C | NM_001136552.1^a^ |
|  |  | R: GGTGTCACCCCATTTGCATG |  |  |  |
|  | *palld* | F: ACACATGTCGAGTGCTCTGA | 104 | 59˚C | BT059828.1^a^ |
|  |  | R: TGTCTGACAACAACCATGGATG |  |  |  |
|  | *ap1m2* | F: CTACCGCATCAACACTCACG | 134 | 59˚C | BT060364.1^a^ |
|  |  | R: CCACGTTGTTGGCTACTGAC |  |  |  |
|  | *arsb* | F: GGTGCAACAAAACTCAAATCAGG | 167 | 59˚C | NM_001173652.1^a^ |
|  |  | R: CGAAAGGTTGCTGGTTCGAA |  |  |  |
|  | *litaf* | F: ACTCTTGCAGTCGTTCCCTC | 134 | 59˚C | BT056544.1^a^ |
|  |  | R: CAACTGGGCAGGATGTGAAA |  |  |  |
|  | *g3bp* | F: GCAAAGGAACCGAGAAGTCC | 189 | 59˚C | BT059216.1^a^ |
|  |  | R: CATCTCCACGGTCAAACAGC |  |  |  |
|  | *cfl2* | F: AGCCTATGACCAACCCACTG | 224 | 60˚C | TC63899^b^ |
|  |  | R: TGTTCACAGCTCGTTTACCG |  |  |  |
|  | *ef1a* | F: CTGCCCCTCCAGGACGTTTACAA | 175 | 60˚C | AF321836^a^ |
|  |  | R: TGTTCACAGCTCGTTTACCG |  |  |  |
| *PCR* | *nptII* | F: CTCACCTTGCTCCTGCCGAGA | 215 | 60˚C | KJ081792.1^a^ |
|  |  | R: CGCCTTGAGCCTGGCGAACAG |  |  |  |
|  | *gh* | F: GGTCCTGAAGCTGCTCCATA | 223 | 59˚C | LOC100136588^a^ |
|  |  | R: CCTTGGGGTTTACAGTGCAC |  |  |  |

^a^ GenBank ([http://www.ncbi.nlm.nih.gov](http://www.ncbi.nlm.nih.gov/))

^b^ Atlantic salmon Gene Index (http://compbio.dfci.harvard.edu/tgi)

*fads2d6*, delta-6 fatty acyl desaturase; *fads2d5*, delta-5 fatty acyl desaturase; *elovl2*, fatty acyl elongase 2; *elovl5a*, fatty acyl elongase 5 isoform a; *elovl5b*, fatty acyl elongase isoform b; *palld*, pallidin; *ap1m2*, AP-1 complex subunit mu-2; *arsb*, arysulfatase B; *litaf*, lipopolysaccharide-induced tumor necrosis factor-alpha factor homolog; *g3bp*, galectin-3-binding protein; *nptII*, neomycin phosphotransferase II; *gh*, growth hormone
